# Melatonin strongly enhances the *Agrobacterium*- mediated transformation of carnation in nitrogen-depleted media

**DOI:** 10.1186/s12870-023-04325-5

**Published:** 2023-06-14

**Authors:** Omid Aalami, Pejman Azadi, Hanieh Hadizadeh, H. Dayton Wilde, Zahra Karimian, Hossein Nemati, Leila Samiei

**Affiliations:** 1 Research Center for Plant Sciences, Ferdowsi University of Mashhad, Mashhad, 91779948974 Iran; 2 Department of Horticultural Science and Landscape, Faculty of Agriculture, Ferdowsi University of Mashhad, Mashhad, 91779948974 Iran; 3 Department of Genetic Engineering, Agricultural Biotechnology Research Institute of Iran (ABRII), Agricultural Research, Education and Extension Organization (AREEO), Tehran, Iran; 4 Horticulture Department, University of Georgia, Athens, GA USA

**Keywords:** *Agrobacterium tumefaciens*, Antioxidant compounds, *Dianthus caryophyllus*, α-lipoic acid, Transformation efficiency

## Abstract

With the rising demand for new cultivars of carnation, efficient transformation protocols are needed to enable the bioengineering of new traits. Here, we established a novel and efficient *Agrobacterium*-mediated transformation system using callus as the target explant for four commercial carnation cultivars. Leaf-derived calli of all cultivars were inoculated with *Agrobacterium tumefaciens* strain LBA4404 containing the plasmid pCAMBIA 2301 harboring genes for β-glucuronidase (*uidA*) and neomycin phosphotransferase (*npt*II). Polymerase chain reaction (PCR) and histochemical assays confirmed the presence of *uidA* and β-glucuronidase (GUS), respectively in transgenic shoots. The effect on transformation efficiency of medium composition and the presence of antioxidants during inoculation and co-cultivation was investigated. The transformation efficiency was increased in Murashige and Skoog (MS) medium lacking KNO_3_ and NH_4_NO_3,_ and also in MS medium lacking macro and micro elements and Fe to 5% and 3.1% respectively, compared to 0.6% in full-strength medium. Transformation efficiency was increased dramatically to 24.4% across all carnation cultivars by the addition of 2 mg/l melatonin to nitrogen-depleted MS medium. Shoot regeneration was also doubled in this treatment. The establishment of this efficient and reliable transformation protocol can advance the development of novel carnation cultivars through molecular breeding approaches.

**Supplementary Information:**

The online version contains supplementary material available at 10.1186/s12870-023-04325-5.

## Highlights

Melatonin at 2 mg/l substantially increases the *Agrobacterium*-mediated transformation efficiency of carnation cultivars to 24%.

Nitrogen-depleted inoculation and co-cultivation media can greatly enhance carnation transformation.

Callus is a potential target explant for *Agrobacterium* transformation of carnation.

Melatonin and lipoic acid can considerably promote the shoot regeneration from transformed callus.

The established protocol for carnation transformation is cultivar independent.

**Supplementary Information:**

The online version contains supplementary material available at 10.1186/s12870-023-04325-5.

## Background

Carnation (*Dianthus caryophyllus* L.), with rich flower color and form, represents one of the major floricultural crops with high ornamental and commercial value throughout the world. Numerous carnation cultivars around the world have been bred for the desirable and novel characteristics such as new flower color, shape and size, improved fragrance, disease resistance, higher productivity, and longer vase life [1]. Given the ever-increasing demands for new features in ornamental plants including carnation, the establishment of a reliable genetic transformation system is an area of high priority.

*Agrobacterium*-mediated transformation is one of the popular genetic transformation methods, particularly in dicotyledonous plant species, in which a gene of interest is transferred to a host plant through T-DNA (transferred DNA) of *Agrobacterium tumefaciens.* The activation of the virulence genes (*vir* genes) in *Agrobacterium* and the appropriate bacterial attachment to the host cells are the crucial steps that ensure the success of transformation procedure [2]. Several factors including the strain of *Agrobacterium*, plant genotype, the source and age of explant, inoculation and co-cultivation medium, selection pressure and regeneration protocol are involved in the success of *Agrobacterium*-mediated transformation [3–5].

Carnation transformation using *Agrobacterium* has been practiced in several investigations, most of which reported rather low transformation efficiency (less than 10%) [6–10]. However, a lack of a transformation system with a high rate of cultivar-independent gene transfer has been remained a principal constraint to the further molecular breeding of this species.

The type of the target explant is one of the determining factors in the success of transformation system since the regeneration competency of cell types, tissues and organs varies considerably following infection with *A. tumefaciens* [11, 12]. So far, explants including internodes, leaves, and cotyledons [13–15], stems [16], and petals [6, 17] have been used as target explant for carnation transformation. Callus has not been utilized in carnation transformation, likely due to challenges encountered during callus regeneration [18]. Callus, either organogenic or embryogenic has been used frequently for the *Agrobacterium* transformation of many plant species with considerable success [19–21]. In the present study, we investigated callus as an explant for transformation of multiple carnation cultivars.

Apart from explant type, the composition of inoculation and co-cultivation media has been identified as a crucial factor in transformation efficiency. In some studies, reducing MS medium salts was reported to facilitate transfer of T-DNA to the host cells. For example, reducing MS medium strength enhanced transformation in Wheat, Canola and Cucumber [22–24]. Other studies indicated that the omission of certain elements from medium had positive influence in *Agrobacterium* transformation. The elimination of KH_2_PO_4_, NH_4_NO_3_, KNO_3_ and CaCl_2_ from inoculation and co-cultivation media enhanced transformation in Lilium, Petunia and Tall fescue, considerably [25–27]. In carnation, the use of filter paper soaked only with water and/or acetosyringone rather than the phytohormone rich MS medium is suggested for achieving higher transformation rate [13]. However, the alternative strategy regarding the elimination of a number of components from nutrient media of inoculation and co-cultivation has not been attempted so far. Therefore, we hypothesize that the manipulation of MS medium composition and omission of some individual components from inoculation and cocultivation media might have positive influence on the efficiency of *Agrobacterium* transformation of carnation.

A vigorous regeneration of shoots or somatic embryos from explants following co-cultivation is critical for the success of plant transformation. The explants’ loss of vigor and browning after exposure to *Agrobacterium* often seems to be the main reasons for the poor transformation efficiency. *Agrobacterium tumefaciens* is a plant pathogen which can cause the death of target plant tissues following transformation in many plant species [28]. The initial plant defense response to the invasion of pathogens is associated with the generation of Reactive Oxygen Species (ROS) [29]. The accumulation of ROS can cause cell damage and retarded growth leading to the reduced regeneration and transformation [30]. Antioxidants are known to mitigate the detrimental effects of ROS in plants and are reported to be beneficial in *Agrobacterium* mediated transformation either by alleviating explant browning or promoting plant regeneration [31–33]. Melatonin and α-lipoic acid (LA) are amongst the chemical compounds with known antioxidant activity. These compounds have been used effectively in studies to promote the stable transformation and reduce cell death and tissue browning in tomato, wheat, cotton and soybean [34, 35]. In the present study, the role of these compounds will be examined for the first time for carnation transformation.

The objective of the present study is to improve genetic transformation of carnation by investigating two strategies: (1) the removal of major elements from the inoculation and co-cultivation media and (2) the addition of the antioxidants melatonin and LA. The novel protocol established in the present study could contribute developing new cultivars with desirable features and facilitate future genetic transformation and breeding program in carnation.

## Results

### Effect of kanamycin on callus survival

This experiment was performed in order to determine the optimized level of kanamycin for carnation calli in selection medium. In this medium, the transformed calli remain intact due to possessing *npt*II genes which confer resistance to kanamycin, while non-transformed calli die in the presence of kanamycin. The results showed that with the increase in concentration of kanamycin in MS medium, callus survival rate was dramatically decreased in all cultivars (Table 1). No callus survived when 200 mg/l kanamycin was applied. Kanamycin at 150 mg/l resulted in a 10% survival rate with all cultivars and, therefore, this level was subsequently used for selection in transformation experiments. It should be noted that any of the survived callus initiated shoot regeneration till the end of the third week which was the end of the experiment.

**Table 1:** The effect of various concentrations of kanamycin on callus survival rate of four carnation cultivars

Kanamycin concentration (mg/l)	Survival rate (%)			
	White Liberty	Tabasco	Cameron	Noblesse
0	100.00 ± 0.00 a	100.00 ± 0.00 a	100.00 ± 0.00 a	100.00 ± 0.00 a
25	80.00 ± 3.50 b	85.00 ± 2.80 b	80.00 ± 0.00 b	90.00 ± 2.04 a
50	65.00 ± 4.5 c	65.00 ± 2.04 c	60.00 ± 2.04 c	70.00 ± 3.50 b
100	30.00 ± 2.04 d	30.00 ± 4.08 d	25.00 ± 2.04 d	35.00 ± 3.50 c
150	10.00 ± 2.04 e	10.00 ± 2.88 e	10.00 ± 0.00 e	10.00 ± 2.04 d
200	0.00 ± 0.00 e	0.00 ± 0.00 e	0.00 ± 0.00 f	0.00 ± 0.00 d

The values represent the mean ± SD of 4 independent measurements. The different letters indicate significant differences (*P* ≤ 0.05) as determined by LSD test.

### Effect of the composition of inoculation and co-cultivation media on transformation

#### Effect of medium composition on callus transformation

**Fig. 1:**
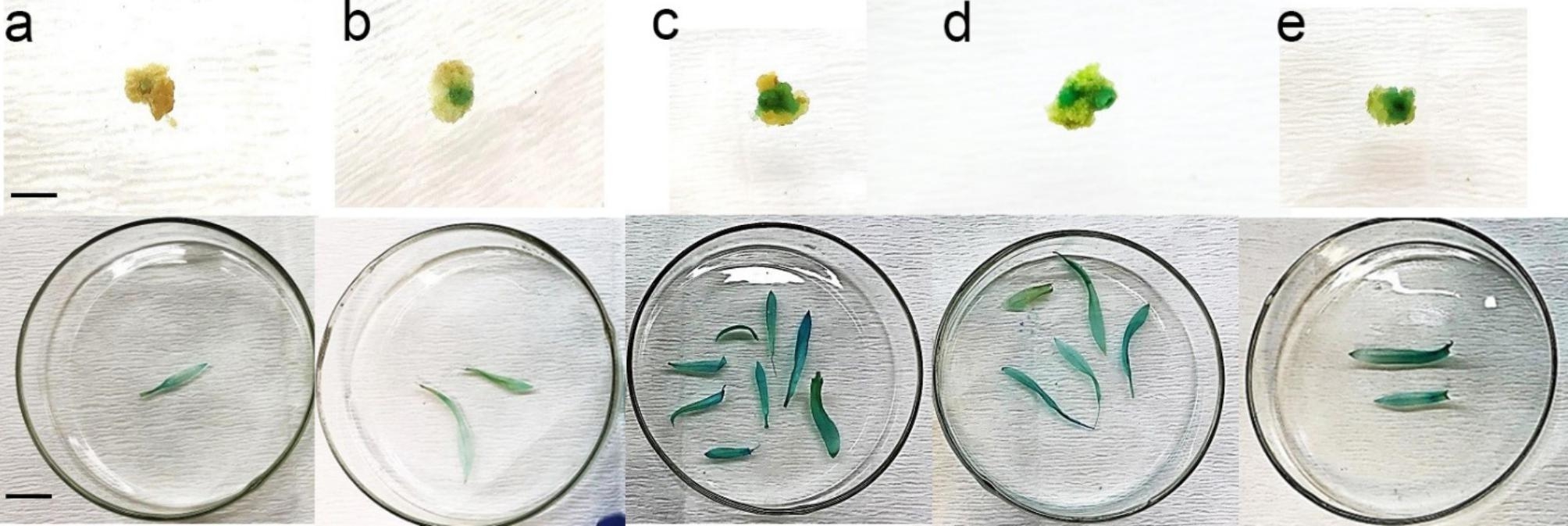
Histochemical GUS assay of callus and leaves of transgenic shoots regenerated from transformed calli in modified inoculation and co-cultivation media. ***a***: callus explants inoculated with *A. tumefaciens* and the leaf obtained from the shoots regenerated in MS01 (control), ***b***: MS02, ***c***: MS03, ***d***: MS04 and ***e***: MS05 media during gene transformation (bars are equal to 1 and 1.25 cm in callus and leaf, respectively)

The effect of media composition of inoculation and co-cultivation media on T-DNA transfer was determined by measuring GUS expression histochemically seven days after exposure to *Agrobacterium* (Fig. 1). The number of GUS spots was minimal on explants cultured in full-strength MS (15.37 spots/100 mg) compared to the rest of media (Table 2). Explants cultured in MS05 exhibited a considerable increase in the number of GUS spots, reaching to 51.81 spots per 100 mg calli. The number of GUS spots also increased significantly in MS03 (45.93 spots/100 mg) and MS04 (43.50 spots/100 mg). All cultivars displayed similar patterns in terms of GUS staining and no GUS activity was observed in non-transgenic calli. Our results confirmed the successful delivery and expression of *uidA*.

**Table 2:** The effect of various compositions of inoculation and co-cultivation media on *Agrobacterium*-mediated transformation of carnation

Inoculation and co-cultivation medium	No of GUS spot per 100 mg callus	Kanamycin- resistant calli (%)	No. of transformed shoots	Transformed shoot regeneration (%)	Gen transformation (%)
Full MS medium (MS01)	15.37 ± 0.71 d	12.81 ± 2.03 b	0.5 ± 0.18 c	3.75 ± 1.25 a	0.62 ± 0.62 b
MS without Macro elements (MS02)	20.93 ± 0.83c	16.56 ± 2.76 b	0.81 ± 0.22 bc	5.62 ± 1.28 a	1.25 ± 0.85b
MS without KNO_3_ and NH_4_NO_3_ (MS03)	45.93 ± 1.08 b	22.81 ± 3.06 a	1.93 ± 0.35 a	10.00 ± 2.04 a	5.00 ± 1.29 a
MS without Macro, Micro elements, and Iron (MS04)	43.50 ± 1.31 b	19.68 ± 2.86 a	1.37 ± 0.27 ab	10.00 ± 2.04 a	3.12 ± 1.19 ab
MS without Macro, Micro elements, Iron and Vitamin (MS05)	51.81 ± 1.12 a	9.37 ± 1.81 c	1.12 ± 0.22 bc	6.87 ± 1.19 a	1.25 ± 0.85 b

The values represent the mean ± SD of 16 values in four carnation cultivars (The value for each cultivar is the average of 40 independent measurements). The different letters indicate significant differences (*P* ≤ 0.05) as determined by LSD test.

The number of GUS positive per 0.1 g calli was scored 7 days after bacterial inoculation. The percentage of kanamycin-resistant calli was obtained after 3 months of selection culture.

The effect of various compositions of inoculation and co-cultivation media on production of kanamycin-resistant calli is shown in Table 2. The highest rate of putative transgenic callus was obtained in medium lacking KNO_3_ and NH_4_NO_3_ (MS03), while the smallest number of calli were produced on MS05, which lacked micro- and macronutrients, Fe, and vitamins. Carnation cultivars differed in the rate of production of kanamycin-resistant calli (Table 3). Averaged across all media, the rate of kanamycin-resistant calli was significantly higher in ‘Noblesse’ (25.25%) and ‘Tabasco’ (25.00%) while it was lowest in ‘Cameron’ (4.50%). The interaction between various media with different carnation cultivars was not significantly different in terms of percentage of kanamycin-resistant calli (Supplementary file S1).

**Table 3:** The rate of kanamycin-resistant calli in various carnation cultivars under the effect of modified inoculation and co-cultivation media

Cultivar	Kanamycin-resistant calli (%)
White Liberty	10.25 ± 1.11 b
Tabasco	25.00 ± 1.73 a
Cameron	4.50 ± 1.00 c
Noblesse	25.25 ± 1.67 a

The values represent the mean ± SD of twenty independent measurements. The different letters indicate (*P* ≤ 0.05) as determined by LSD test.

#### Effect of medium composition on transformed shoot regeneration

The number of regenerated shoots and shoot regeneration percentage were not affected by different cultivars in this assay. Therefore, the data depicted in Table 2 are the average data of four cultivars. The total number of regenerated shoots was significantly greater on MS medium lacking KNO_3_ and NH_4_NO_3_ (MS03) and in MS medium devoid of macro- and micro elements and Fe (MS04). The modified MS media were not significantly different from each other in terms of shoot regeneration rate (calculated as transformed shoot over the transformed callus number). Nevertheless, the maximum transformed shoot regeneration (10.00%), averaged among all cultivars, was recorded in MS03 and MS04 (Table 2). Full-strength MS medium (MS01) showed the minimum regeneration percentage (3.75%). A significant increase in transformation frequency was observed in MS medium lacking KNO_3_ and NH_4_NO_3_ (5.00%), which was eight times higher than that in full-strength MS medium (0.62%). Elimination of macro- and micro elements as well as Fe from MS medium (MS04) also showed better results than control in terms of gene transformation efficiency (3.12%). No significant differences were observed among cultivars (Supplementary file S2).

### PCR analysis of transgenic carnations from media composition experiments

**Fig. 2:**
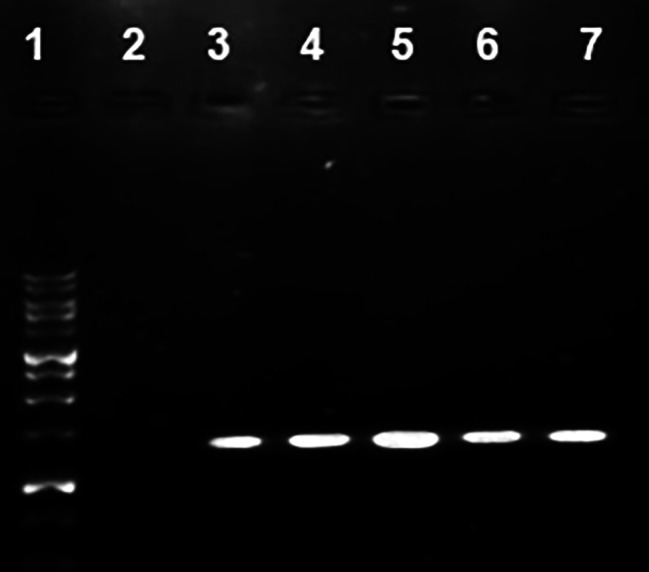
PCR positive shoots recovered from all media. Lane ***1***, molecular size marker; Lane ***2***, negative control (non-transformed plant); Lanes (***3–7***) amplification of 1400 bp fragment of GUS gene in putative transgenic carnations in all media. Full-length gel is included in the supplementary file (S3)

GUS expression was observed in the leaves of regenerated shoots three months after inoculation (Fig. 1). In order to confirm the presence of *uidA* in the carnation genome, PCR amplification was performed using total genomic DNA from all putatively transformed shoots that survived kanamycin treatment. PCR analysis showed the amplification from all transgenic carnation cultivars of the 1400 bp DNA fragment expected from the GUS gene (Fig. 2). No band was detected in the non-transformed control. PCR-positive shoots were recovered from all of the five media examined.

### Effect of antioxidant compounds on ***Agrobacterium***-mediated transformation in carnation

#### Effect of antioxidants on callus transformation

**Fig. 3:**
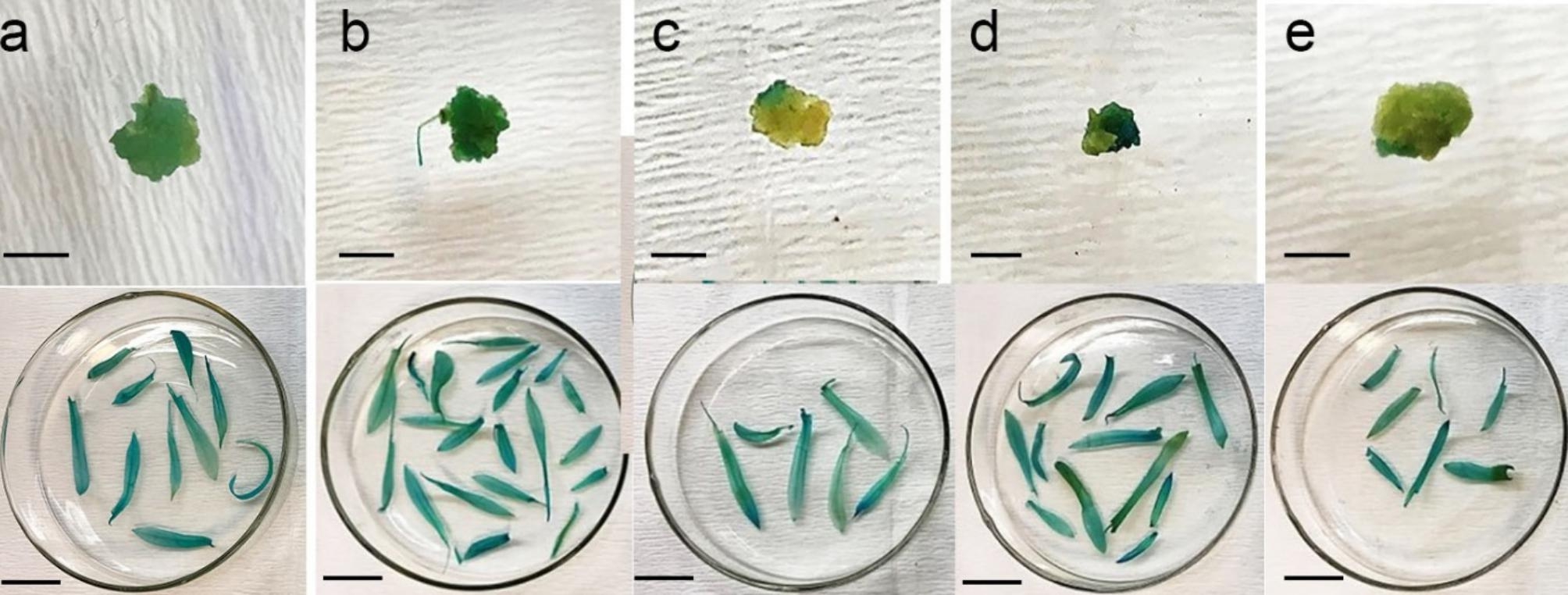
Histochemical GUS assay of callus and leaves of transgenic shoots regenerated from transformed calli affected by supplementation of Melatonin and LA in inoculation and co-cultivation media. ***a***: callus explants inoculated with *A. tumefaciens* and the leaves obtained from the shoots regenerated from transformed callus in MS03 (control) ***b***: 2 mg/l melatonin, ***c***: 20 mg/l melatonin, ***d***: 2 mg/l α-lipoic acid, and ***e***: 20 mg/l α-lipoic acid during gene transformation (bars are equal to 1 and 1.5 cm in callus and leaf, respectively

Supplementation of MS03 medium with 2 mg/l melatonin resulted in the highest number of GUS spots on calli (89.43 spots/100 mg) (Fig. 3; Table 4). LA at 2 mg/l also enhanced the number of GUS positive calli (75.06). GUS activity was not observed in non-transgenic calli. High level of LA and melatonin (20 mg/l) resulted in no increase or significant decrease in GUS spots, respectively, compared to control (MS03). No significant differences were observed amongst cultivars regarding the stable GUS expression on calli of transgenic plants.

**Table 4:** The effect supplementation of inoculation and co-cultivation media with melatonin and α-lipoic acid on *Agrobacterium*-mediated transformation of carnation

Inoculation and co-cultivation medium	No. of GUS spot per 100 mg callus	Kanamycin- resistant calli (%)	No. of transformed shoots	Transformed shoot regeneration (%)	Gene transformation (%)
MS without KNO_3_ and NH_4_NO_3_ (Control)	60.25 ± 1.88 c	24.37 ± 1.87 a	1.93 ± 0.42 b	9.37 ± 1.92 b	5.62 ± 1.28 bc
Melatonin 2 mg/l	89.43 ± 1.48 a	30.00 ± 1.76 a	4.68 ± 0.43 a	18.75 ± 1.54 a	24.37 ± 2.73 a
Melatonin 20 mg/l	31.06 ± 1.44 d	10.93 ± 2.10 b	0.62 ± 0.15 c	5.62 ± 1.28 b	2.50 ± 1.11 c
α-lipoic acid 2 mg/l	75.06 ± 1.63 b	25.62 ± 2.03 a	2.06 ± 0.26 b	13.12 ± 1.19 a	9.37 ± 1.70 b
α-ipoic acid 20 mg/l	56.87 ± 2.10 c	11.56 ± 2.07 b	0.87 ± 0.20 c	6.25 ± 1.25 b	3.75 ± 1.25 c

The values represent the mean ± SD of 16 independent measurements in four carnation cultivars. The different letters indicate significant differences (*P* ≤ 0.05) as determined by LSD test. The number of GUS positive per 100 mg calli was scored 7 days after bacterial inoculation. The percentage of kanamycin-resistant calli was obtained after 3 months of selection culture.

The supplementation of inoculation and co-cultivation media with 2 mg/l melatonin or LA increased kanamycin-resistant callus production slightly, but not significantly, compared to MS03 medium alone (Table 4). Higher levels of melatonin and LA (20 mg/l) dramatically decreased the frequency of kanamycin-resistant calli. All cultivars responded similarly with respect to transformation frequency in the presence of the antioxidants.

#### Effect of antioxidants on transformed shoot regeneration

**Fig. 4:**
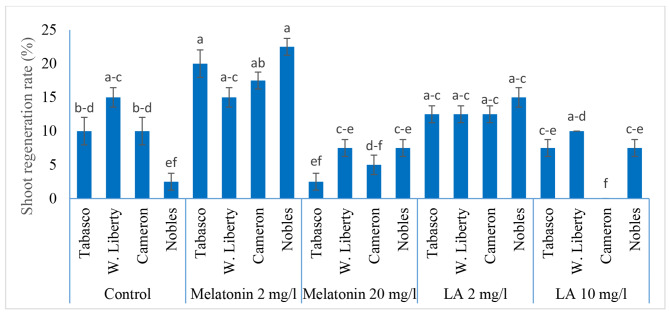
The effect of supplementation of melatonin and LA in inoculation and co-cultivation media on transformed shoot regeneration rate in various carnation cultivars

The regeneration rate of transformed shoot was affected by both carnation cultivars and antioxidant type and level in inoculation and co-cultivation media. Melatonin and LA at 2 mg/l considerably enhanced shoot regeneration, respectively (18.75%, 13.12%) compared to control (9.37%) (Table 4). The minimum shoots were regenerated on medium supplementing with 20 mg/l melatonin without significant difference with medium containing 20 mg/l LA and control. ‘Noblesse’ cultivar gave rise to the highest shoot regeneration rate in medium containing 2 mg/l melatonin, while it produced the least shoot regeneration rate in control. The maximum and minimum shoot regeneration rate for ‘Tabasco’, was obtained in medium combined with 2 and 20 mg/l melatonin, respectively (Fig. 4).

The highest shoot number (4.68) was obtained in the medium supplemented with 2 mg/l melatonin without significant different with control (1.93) (Table 4). However, medium containing 20 mg/l melatonin produced the least number of shoot (0.62) without significant difference with medium possessing 20 mg/l LA (0.87). Melatonin and LA, therefore, tend to represent remarkable responses in carnation shoot regeneration only when they are used at low levels in inoculation and co-cultivation media.

Gene transformation efficiency was also affected using various concentration of melatonin and LA in inoculation and co-cultivation media. Our findings suggested a significant 4.33-fold increase in gene transformation efficiency from 5.62% in nitrogen-depleted control to 24.37% in media supplemented with 2 mg/l melatonin. Interestingly, this was even 39.30-fold higher than gene transformation efficiency in full-strength MS media. LA at 2 mg/l also enhanced gene transformation frequency to 9.37%, yet this was not significantly different from nitrogen-depleted control. Melatonin and LA at 20 mg/l showed negative impact on the rate of transformation compared to control. Carnation cultivars were not significantly different with each other in terms of gene transformation rate in various media (Supplementary file S4).

### PCR analysis of transgenic shoots from antioxidant treatments

**Fig. 5:**
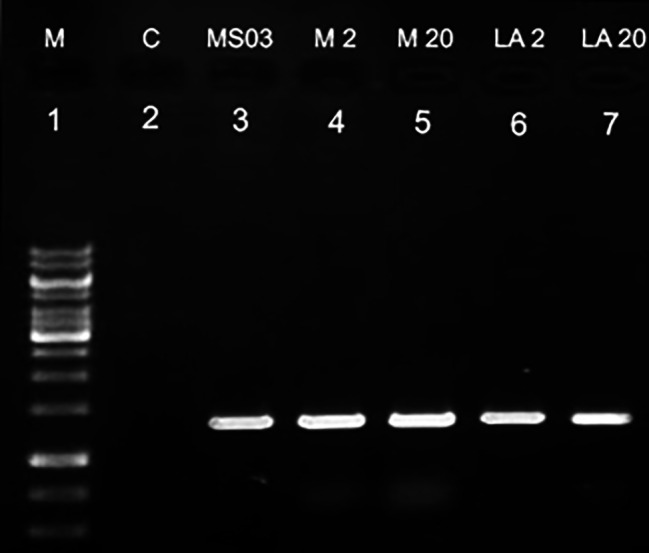
PCR positive shoots recovered from all media. Lane ***1***, molecular size marker; Lane ***2***, negative control (non-transformed plant); Lanes (***3–7***) amplification of 1400 bp fragment of GUS gene in putative transgenic carnations in all media. Full-length gel is included in the Supplementary file (S5)

Amplification of a 1400 bp product with GUS gene primers confirmed transgene integration into all four cultivar representatives. Figure 5 shows the amplification of the GUS gene of five selected shoots from each cultivar. No band was detected in negative control.

## Discussion

Previous studies reported low transformation efficiency in carnation using several approaches [6, 7, 10]. In the present study, we significantly improved the transformation efficiency of four carnation cultivars. This was done for the first time using the novel antioxidant compounds or modified MS medium composition in inoculation or co-cultivation media.

Prior to establishment of a transformation system, an efficient selection process should be determined [17, 36]. The most widely used marker for selection of transgenics is resistance to the antibiotic kanamycin which is conferred by a gene encoding the enzyme neomycin phosphotransferase ΙΙ (*npt*ΙΙ). Excessive concentration of Kanamycin in selection media can hamper explant regeneration and lead to necrosis of transformed tissues while insufficient doses may increase the number of escape explants which reduce transformation efficiency [37]. The optimal level of kanamycin for the target plants, therefore, need to be carefully determined as it may vary greatly among or even within species [38]. Kanamycin-resistant calli of carnation are green and can easily be distinguished from kanamycin-sensitive calli which have generally pale-yellow color. In the present study, the response of all four carnation cultivars was similar and therefore 150 mg/l of kanamycin was determined as the optimal selection concentration for all cultivars.

The use of callus in our study as target explant for *Agrobacterium* transformation of carnation was highly successful, with a high transformation rate being obtained. There are several reports of improved transformation efficiency using callus as the target explant in other species like *Lilium longiflorum, Ornithogalum dubium*, and *Camellia sinensis*, indicating that undifferentiated cells with rapid division rate could be considered as viable tissue for genetic transformation [39, 40]. Based on the favorable results of the present study, callus was an appropriate explant for carnation transformation system.

To determine the optimal medium for transformation of carnation with *A. tumefaciens*, various compositions of MS-based inoculation and co-cultivation media were evaluated. Our results indicated that the elimination of KNO_3_ and NH_4_NO_3_ from inoculation and co-cultivation media (MS03) substantially increased the regeneration response and transformation efficiency by 166% and 700%, respectively, compared to full-strength MS medium. This considerable increase, particularly in gene transformation rate, suggests that either reducing K concentration or removing nitrogen source from medium enhances *Agrobacterium* transformation to the target plant. This can affect the attachment of *Agrobacterium* to the explants as well as its clustering characteristics which then determine the transformation efficiency [13]. In accordance with the findings of this study, the elimination of NH_4_NO_3_ from co-cultivation medium enhanced transformation in Lilium. This was suggested to be due to the inhibitory effects of NH_4_NO_3_ on the proliferation of *Agrobacterium* [41]. Moreover, in another study on Lilium, MS medium free of NH_4_NO_3_, KNO_3_, and CaCl_2_ improved transformation efficiency to 25.4% [25]. In *Eustoma grandiflorum*, 4 to 8 times higher expression frequency was observed when nitrogen-depleted medium was used for co-cultivation [42]. Furthermore, Sharafi et al. indicated that removal of NH_4_NO_3,_ KH_2_PO_4_ and KNO_3_ significantly increased the transformation efficiency of *Papaver bracteatum* to 34.3% [43].

Reduction of the K concentration beside the removal of nitrogen source could also be responsible for the enhanced transformation in MS03 media. Similar results were already reported in transformation of a number of plant species [25, 26, 43]. In a recent study on Tall fescue transformation, the presence of both K and N ions in inoculation and co cultivation medium had negative effect on transformation. It was suggested that these ions might reduce *Agrobacterium* proliferation and attachment to the explant [27]. This is, however, in contrast to the findings of Boyko et al. (2009) who reported enhanced Homologous recombination frequency (HRF) and transformation in *Arabidopsis thaliana* with the increase in K concentration in culture medium [44]. It is evident that the actual role of K on *Agrobacterium* transformation and its relevant mechanism is still unclear and require further investigation.

MS medium lacking macro, micro and Fe (MS04) also showed higher regeneration and transformation rates compared to the full-strength control. This is consistent with the findings of Dupre et al. (2000) who reported the enhancement in gene transformation rate of *Ginkgo biloba* using co-cultivation medium lacking several mineral components [45]. Furthermore, in a review published by Opabode (2006), the use of reduced salt inoculation and co-cultivation media was proposed to be beneficial for enhancing T-DNA delivery, transient GUS expression and transformation efficiency in several species [23]. In our study, all treatments with two or more components omitted from MS medium showed better results in terms of GUS expression and transformation rate than the original MS medium, suggesting that macro- and micro elements, Fe or even vitamins in inoculation and co-cultivation media could have inhibitory effects on *A. tumefaciens* mediated transformation of carnation.

Transformation efficiency was further optimized by investigating two antioxidant compounds in inoculation and co-cultivation media. The supplementation of inoculation and co-cultivation media with 2 mg/l melatonin dramatically enhanced transformation by 4.3-fold compared to nitrogen-depleted MS medium and 39.3-fold compared to full-strength MS medium. Melatonin was suggested to enhance the attachment of *Agrobacterium* to the cell surface, thereby enhancing transformation [35, 46]. Also, melatonin was revealed to help maintain normal cell surface in transformed plants. In *Agrobacterium*-mediated transformation of Banana, the transformed cells treated with melatonin had normal cell surface, while non-treated cells showed surface shrinkage [46]. Melatonin can also protect plant cells from oxidative stress caused by free radicals that are generated during *Agrobacterium* transformation [35]. It acts as an antioxidant to increase viability of explant and reduce tissue necrosis [31].

Melatonin apart from its antioxidant activity, can interact with other phyto-hormones to regulate plant growth and development. It has been shown in several studies that melatonin has auxin-like activities [47–49]. In highbush blue berry melatonin could replace indole butyric acid (IBA) in vitro and enhanced shoot morphogenesis [50]. The higher shoot regeneration and transformation rate obtained in our study in melatonin treated calli could also be due to its auxin–like activity which may enhance organogenesis and shoot regeneration. Melatonin is also suggested to be involved in shoot regeneration through calcium signaling [51, 52]. However, the exact mechanism of melatonin in shoot morphogenesis and transformation is remained to be clarified.

Melatonin has been used infrequently in plant transformation studies. Our results indicated that it was highly effective in carnation transformation at low concentration (2 mg/l), but not high concentration (20 mg/l). Similarly, melatonin at 2 mg/l was more advantageous in soybean stable transformation compared to 20 mg/l [35]. This is, however, in contrast to the findings of Tiwari who reported the beneficial effect of melatonin at 23.2 mg/l in the transformation of Banana CV Grand Naine [46]. Therefore, the effective concentration of melatonin in transformation is species dependent and should be determined empirically.

Similarly, LA showed a 66.13% increase in carnation transformation over the control when supplemented at low concentration (2 mg/l) in inoculation and co-cultivation media. This is in line with the findings of previous works indicating LA at low concentration (0.2–2.1 mg/l) enhanced gene transformation efficiency in several species [34, 53]. In contrast, LA was advantageous in higher concentrations (10–100 mg/l) in soybean, Mexican lime, and Lilium [34, 54, 55]. Therefore, the effective level of LA should also be optimized for any untested plant species before transformation. LA is a ubiquitous disulfide antioxidant and is suggested to enhance plant transformation rate through reducing tissue browning, increasing GUS transient expression, and reducing the number of escapes [34, 35].

In the present study, the expression of GUS genes in carnation was significantly increased using LA and melatonin at concentration of 2 mg/l compared to control medium. Low levels of LA (2–4 mg/l) also enhanced transient GUS expression in tomato [34] and soybean [56]. In general, it can be argued that LA and melatonin at low concentration could protect transformed cells or tissues from browning and promote regeneration, thereby enhancing GUS expression in gene transformation [57]. Antioxidants, as anti-necrotic compounds, not only stimulate and facilitate T-DNA delivery, but also promote stable transformation [58]. Thus, in the presence of antioxidants, the *Agrobacterium*-transformed cells or tissues survive during transformation process and, therefore, GUS expression is enhanced.

## Conclusion

In this study we developed a novel, reliable and stable *Agrobacterium*-mediated transformation system for four carnation cultivars. The success of the present study relies on the improvement of *Agrobacterium*-mediated process by increasing explant regeneration and transformation efficiency. The transformation rate dramatically enhanced to 8.1-fold using MS medium free of KNO_3_ and NH_4_NO_3_ in inoculation and co-cultivation phase. The inclusion of 2 mg/l melatonin increased the transformation rate by an additional 4.3-fold. Overall, with the help of these novel strategies, we were able to substantially enhance carnation transformation from 0.6 to 24.4%. In addition to the high transformation efficiency, this method was found to be cultivar independent. We believe that this approach will not only facilitate the molecular breeding and genome editing of carnation but could also be applied for the improvement of transformation in other plant species.

## Methods

### Plant material and bacterial strain

Leaves of in vitro plantlets of four carnation cultivars including ‘Tabasco’, ‘Noblesse’, ‘White Liberty’ and ‘Cameron’ which all provided by Ornamental Plants Research Center (OPRC) in Mahallat (Iran) were used for callus induction. Formal identification of cultivars analyzed in this study was performed by Dr. Hamid Moazzeni at Department of Botany, Research Center for Plant Sciences. Permission of sample collection was gained in accordance with all the relevant institutional guidelines and legislation. The use of plants in the present study complies with international, national and/or institutional guidelines. In vitro derived leaf explants were cultured on MS medium containing 0.2 mg/l 2-4-D and 0.5 mg/l AgNO_3_ and the regenerated calli of all cultivars were used as explants for *A. tumefaciens* inoculation. The composition of media in each transformation stage is displayed in Table 5. Genetic transformation was conducted with *A. tumefaciens* strain LBA4404 containing the binary plasmid pCAMBIA 2301, which harbors the *uidA* gene for the visual marker β-glucuronidase (GUS) and the *npt*II gene for the selectable marker neomycin phosphotransferase II. Both genes were under the control of cauliflower mosaic virus 35 S promoter and the *uidA* gene contained an intron in the coding region to ensure that GUS was expressed only in plant cells. The freeze/thaw method [59] was used for efficient transformation of *A. tumefaciens* with plasmid DNA.

**Table 5:** Media composition in each stage of transformation procedure

Stage of plant development/ transformation	Media composition	
Callus induction	MS medium + 0.2 mg/l 2-4-D, 0.5 mg/l AgNO_3_, 30 g/l sucrose, 8 g/l agar, pH = 5.7	
Bacterial growth	LB medium (10 g/l tryptone peptone, 5 g/l yeast extract, 10 g/l NaCl, pH 7.0), 50 mg/l kanamycin, 100 mg/l rifampicin	
Inoculation	MS medium (according to the corresponding treatment) + 30 g/l sucrose, pH = 5.7	
Co-cultivation	MS medium (according to the corresponding treatment) + 3 mg/l BAP, 30 g/l sucrose, pH = 5.7	
Selection/Shoot regeneration	MS medium + 3 mg/l BAP, 0.3 mg/l NAA, 150 mg/l kanamycin, 400 mg/l cefotaxime, 30 g/l sucrose and 8 g/l agar, pH = 5.7	
Shoot elongation	MS medium + 1 mg/l kinetin, 0.1 mg/l NAA, 150 mg/l kanamycin, 250 mg/l cefotaxime, 30 g/l sucrose, 8 g/l agar, pH = 5.7	

### Kanamycin selection

Since in the present experiment, *npt*II gene was used as a selection marker for kanamycin-resistant calli, it is essential to initially determine the kanamycin resistant threshold in calli of each carnation cultivar prior to transformation. For this, the calli of all cultivars were cultured individually on MS medium [60] supplemented with various concentrations of filter-sterilized kanamycin (0, 25, 50, 100, 150 and 200 mg/l). Four replicates, each involving 10 pieces of callus, were considered for each cultivar. Cultures were kept at 24–26 °C and 16 h photoperiod. Following three weeks of culture, the best level of kanamycin was determined according to its potential to eliminate 90% of callus explants. In fact, callus survival rate was calculated at the end of experiment as the number of dead calli over the total number of calli in each treatment.

### Agrobacterium-mediated transformation

A single colony of *Agrobacterium* strain was grown overnight (about 12 h) in 10 ml liquid Luria broth (LB) medium [61] (10 g/l tryptone peptone, 5 g/l yeast extract, 10 g/l NaCl, pH 7.0) with 50 mg/l kanamycin and 100 mg/l rifampicin as selective antibiotics in a reciprocal shaker (135 cycles/min) at 28 °C for 48 h. The bacteria were harvested by centrifugation at 3500 rpm for 10 min at 4° C, then re-suspended in 10 ml of each MS liquid inoculation media. The absorbance of suspension was adjusted to the required optical density (OD_600_ = 0.5).

The transformation procedure followed in this experiment was based primarily on the method described by Azadi et al. (2010), with some modifications [25]. Inoculation of calli (1-1.5 cm) of all 4 cultivars with *Agrobacterium* was done in a shaker-incubator at low frequency (3500 rpm) for 10 min at 4 °C to ensure that all explants were in appropriate contact with *A. tumefaciens* suspension. Explants immediately dried on sterile filter paper to remove excess bacterial solution. The calli were subsequently transferred to solid co-cultivation medium (without antibiotic) and kept in darkness for three days. The explants were then washed with 250 mg/l cefotaxime to eliminate *Agrobacterium* and blotted on sterile filter paper before transferring to selection and regeneration medium containing 3 mg/l benzyl aminopurine (BAP), 0.3 mg/l naphthalene acetic acid (NAA), 150 mg/l kanamycin, 400 mg/l cefotaxime, 30 g/l sucrose and 8 g/l agar.

When the shoots emerged, the cultures were transferred to shoot elongation media containing 1 mg/l kinetin, 0.1 mg/l NAA, 150 mg/l kanamycin, 250 mg/l cefotaxime, 30 g/l sucrose and 8 g/l agar. Kanamycin selection was carried out for three months with 30-day subculture intervals under 16 h white florescent light (40 µM m^− 2^ s^− 1^) at 24–26 °C. Cefotaxime and kanamycin were used to control bacterial overgrowth and selection of stable transformants, respectively.

### The effect of modified compositions of inoculation and co-cultivation media on carnation transformation

The purpose of this experiment was to optimize the composition of MS medium to enhance *Agrobacterium* transformation efficiency in four carnation cultivars. In this experiment various components of MS medium including KNO_3_, NH_4_NO_3_, the entire macro- and micro elements, Fe and vitamins were eliminated from inoculation and co-cultivation medium. Full-strength MS medium served as the control (Table 6). It should be noted that the composition of inoculation and co-cultivation media was entirely identical in each treatment except for the co-cultivation medium which was solidified with agar. Genetic transformation rate (%) was calculated by the division of the number of regenerated transformed shoot per the number of inoculated calli *100. The step-by-step images of transformation procedure are provided in supplementary file (S6).

**Table 6:** The composition of MS medium in inoculation and co-cultivation media for carnation transformation

Inoculation and co-cultivation medium	Macro elements					Micro-elements	Fe	Vitamin	Sucrose	Agar	
	NH_4_NO_3_	KNO_3_	CaCl_2_.2H_2_O	KH_2_PO_4_	MgSO_4_.7H_2_O						
MS01*	✓	✓	✓	✓	✓	✓	✓	✓	✓	✓	
MS02	✗	✗	✗	✗	✗	✓	✓	✓	✓	✓	
MS03	✗	✗	✓	✓	✓	✓	✓	✓	✓	✓	
MS04	✗	✗	✗	✗	✗	✗	✗	✓	✓	✓	
MS05	✗	✗	✗	✗	✗	✗	✗	✗	✓	✓	

*MS01 medium contains all the minerals of MS medium.

### The effect of melatonin and α-lipoic acid on carnation transformation

This experiment was performed in order to alleviate oxidative browning of callus tissues as well as enhancing the gene transformation frequency in four cultivars of carnation. Based on the results of the first experiment, MS medium devoid of KNO_3_ and NH_4_NO_3_ represented the highest transformation efficiency in all carnation cultivars, therefore this medium was used as the optimized inoculation and co-cultivation medium in the second experiment. Melatonin and LA were used individually at 2 and 20 mg/l in inoculation and co-cultivation media. The inoculation and co-cultivation media received similar levels of the same antioxidant in each treatment; subsequent transformation procedures followed those of the first experiment.

### Histochemical GUS assay

GUS assays were performed on calli seven days after inoculation using 5-bromo-4-chloro-3-indolyl β-D-glucuronidase (X-Gluc) solution [62]. The number of blue spots, which represented GUS expression, were counted under a stereomicroscope. This assay was repeated on kanamycin-resistant calli, as well as the leaves from regenerated shoots three months after inoculation. Leaves of non-transgenic shoots were used as controls. The leaves or calli were incubated in 50 mM sodium phosphate buffer (pH 7.0) supplemented with 1 mM X-Gluc for 16 h at 37° C in the dark. The tissues were subsequently destained using 50% ethanol to remove background chlorophyll before visualization.

### DNA isolation and PCR analysis

Genetic transformation in regenerated shoots was determined by PCR analysis three months after inoculation with *A. tumefaciens*. Total genomic DNA from young leaves (1 g) of control and putatively transformed shoots was isolated using the cetyltrimethylammonium bromide (CTAB) method [63]. DNA quantity was determined using NanoDrop-Spectrophotometer (NanoDrop Technologies, Wilmington, DE, USA). For PCR amplification of genomic DNA, specific primers of *uidA* were used. Amplifications were performed in 25 µl reaction mixture containing 1 µl of each primer, 0.5 µl dNTPs, 0.2 µl Taq DNA polymerase and 2.5 µl 10X Taq buffer, 17 µl of H_2_O and 2 µl (~ 100 ng) of purified genomic DNA. The PCR was carried out according to the following thermal cycles: 35 cycles of 30 cycles of 94° C for 1 min (denaturation), 56–57° C for 1 min (annealing), 72° C for 2 min (extension), and 72° C for 10 min as final extension. The primer sequences for the PCR amplification of GUS gene were as follows: (5’ TGCGGTCACTCATTACGG 3’) and (5’ CATACCTGTTCACCGACG 3’). Following amplification, 5 µl of each amplified product was separated by electrophoresis on a 1% agarose gel, stained with ethidium bromide and photographed using gel documentation system.

### Statistical analysis

Both experiments were performed based on a completely randomized design with four replications, each consisting of a petri dish with 10–12 pieces of callus. All data were subjected to analysis of variance (ANOVA) test using Minitab software package version 19. The means were compared using LSD test at *P* ≤ 0.05. Arcsine transformation was employed for the standardization of percentage data. Several parameters including percentage of shoot regeneration, number of regenerated shoots, number of GUS spots per 100 mg calli, percentage of kanamycin-resistant calli and gene transfer percentage were examined in both experiments.

## Electronic supplementary material

Below is the link to the electronic supplementary material.

Additional file 1: **S1**-The effect of modified composition of inoculation and co-cultivation media on kanamycin resistant calli (%) of various carnation cultivars.

Additional file 2: **S2**-The effect of modified composition of inoculation and co-cultivation media on gene transformation. rate of various carnation cultivars.

Additional file 3: **S3**-Full length gel images of Fig. 2.

Additional file 4: **S4**-The effect of supplementation of melatonin and LA in inoculation and co-cultivation media on gene transformation rate of various carnation cultivars.

Additional file 5: **S5-**Full length gel images of Fig. 5.

Additional file 6: **S6**-Step by step images of transformation procedure in carnation.

## Data Availability

All data are contained within the manuscript and supplementary materials.
