# Supplementary material for: Melatonin strongly enhances the Agrobacterium- mediated transformation of carnation in nitrogen-depleted media
Source: BMC Plant Biol. 2023 Jun 14;23:316. doi: 10.1186/s12870-023-04325-5 (PMC10265774; doi:10.1186/s12870-023-04325-5)
Supplement: Supplementary file 1 — Additional file 1: S1-The effect of modified composition of inoculation and co-cultivation media on kanamycin resistant calli (%) of various carnation cultivars. [file 12870_2023_4325_MOESM1_ESM.docx]

**S1**-The effect of modified composition of inoculation and co-cultivation media on kanamycin resistant calli (%) of various carnation cultivars
