# Supplementary figures and images for: Melatonin strongly enhances the Agrobacterium- mediated transformation of carnation in nitrogen-depleted media
Source: BMC Plant Biol. 2023 Jun 14;23:316. doi: 10.1186/s12870-023-04325-5 (PMC10265774; doi:10.1186/s12870-023-04325-5)

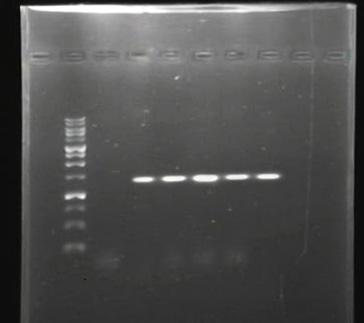


**S3-** **Original gel image of Fig 2:** PCR positive shoots recovered from all media

Supplement: Supplementary file 3 — Additional file 3: S3-Full length gel images of Fig. 2. [file 12870_2023_4325_MOESM3_ESM.docx]

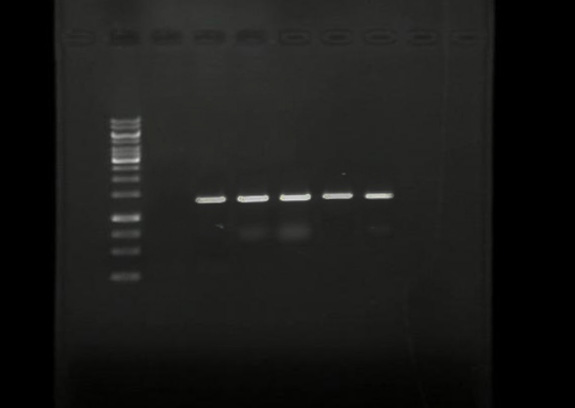


**S5**: **Original gel image of Fig. 5:** PCR positive shoots recovered from all media

Supplement: Supplementary file 5 — Additional file 5: S5-Full length gel images of Fig. 5. [file 12870_2023_4325_MOESM5_ESM.docx]
