# Supplementary material for: Melatonin strongly enhances the Agrobacterium- mediated transformation of carnation in nitrogen-depleted media
Source: BMC Plant Biol. 2023 Jun 14;23:316. doi: 10.1186/s12870-023-04325-5 (PMC10265774; doi:10.1186/s12870-023-04325-5)
Supplement: Supplementary file 4 — Additional file 4: S4-The effect of supplementation of melatonin and LA in inoculation and co-cultivation media on gene transformation rate of various carnation cultivars. [file 12870_2023_4325_MOESM4_ESM.docx]

**S4**-The effect of supplementation of melatonin and LA in inoculation and co-cultivation media on gene transformation rate of various carnation cultivars
