# Supplementary material for: Melatonin strongly enhances the Agrobacterium- mediated transformation of carnation in nitrogen-depleted media
Source: BMC Plant Biol. 2023 Jun 14;23:316. doi: 10.1186/s12870-023-04325-5 (PMC10265774; doi:10.1186/s12870-023-04325-5)
Supplement: Supplementary file 6 — Additional file 6: S6-Step by step images of transformation procedure in carnation. [file 12870_2023_4325_MOESM6_ESM.docx]

**S6**-Step by step images of transformation procedure in carnation

| 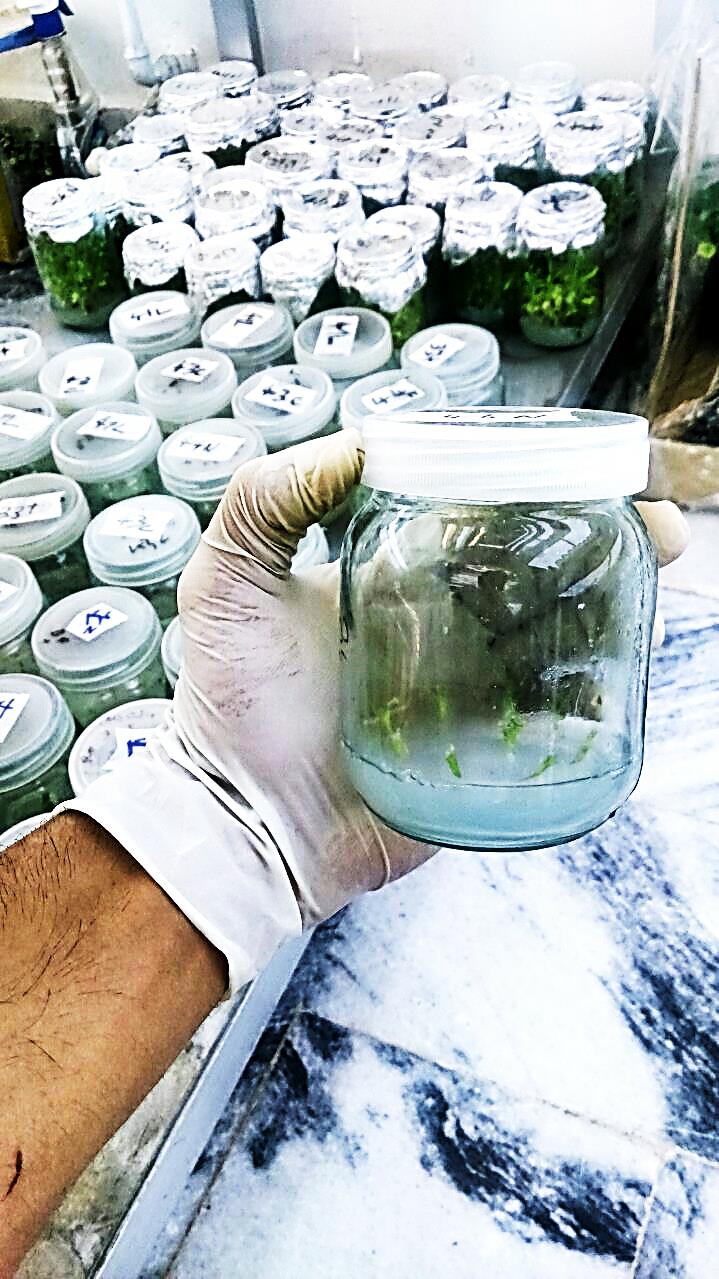 | 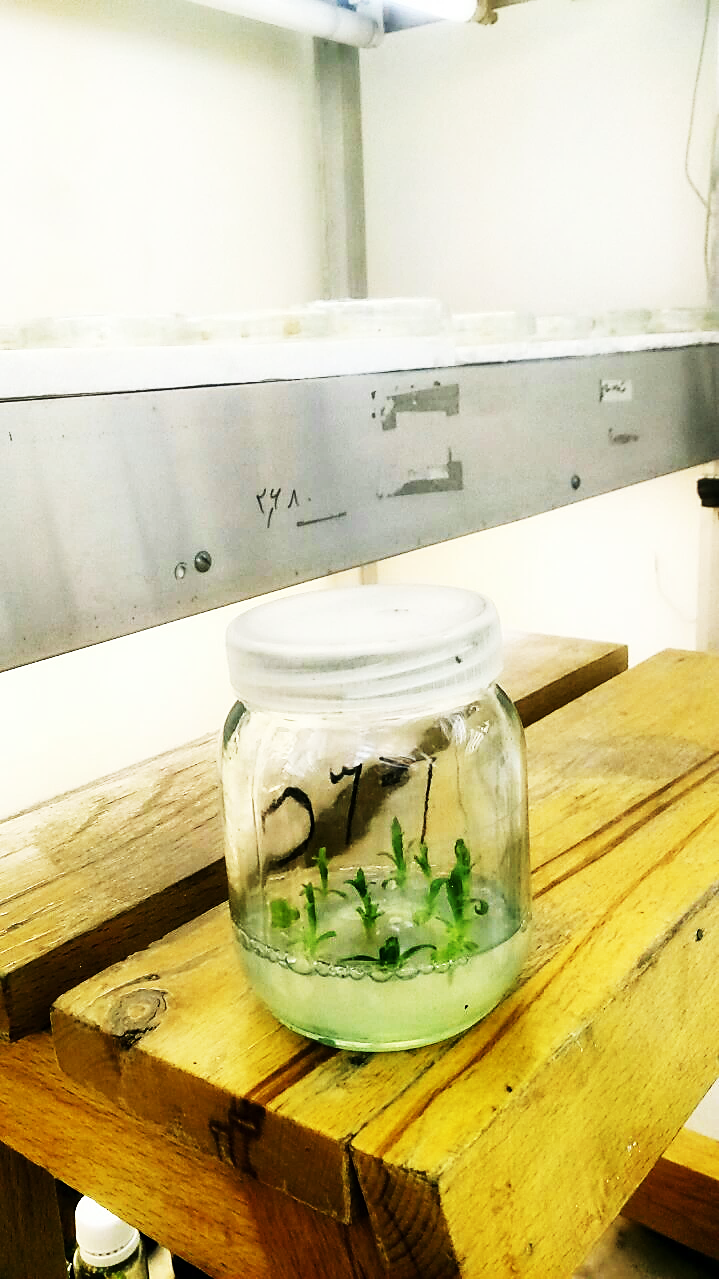 | 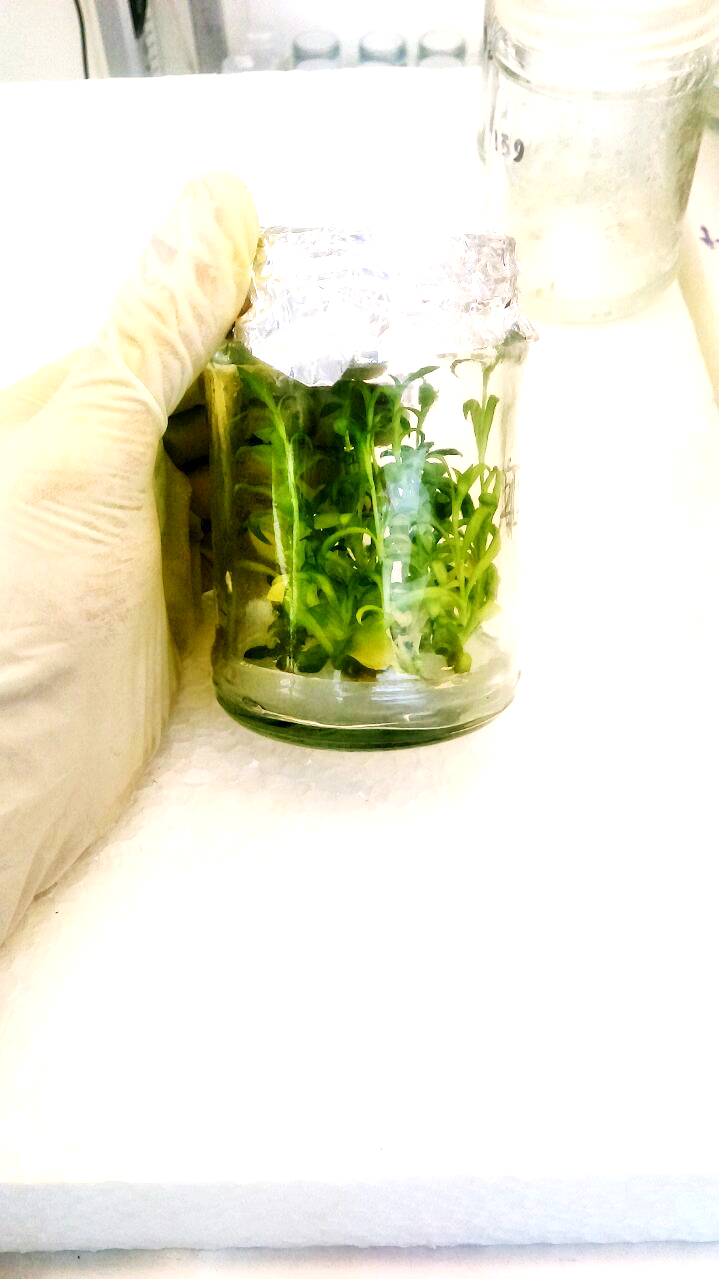 |
| --- | --- | --- |
| Inoculation of carnation axillary buds in proliferation medium to obtain sufficient leaf explants | | |

| **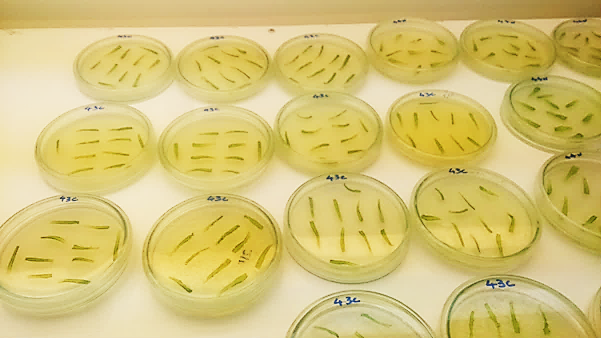** | 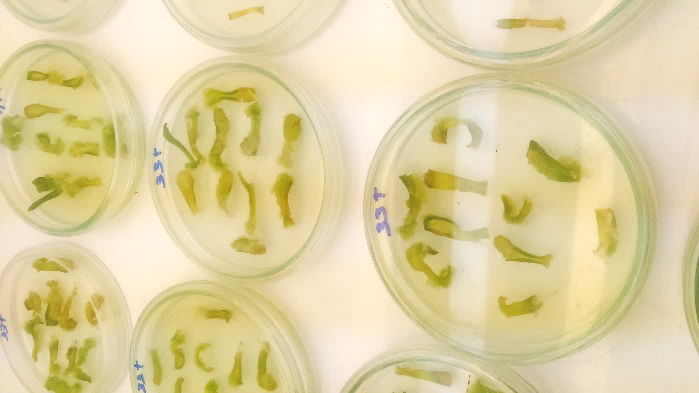 |
| --- | --- |
| Leaf explants in callus induction medium | Induced callus after one month |

| 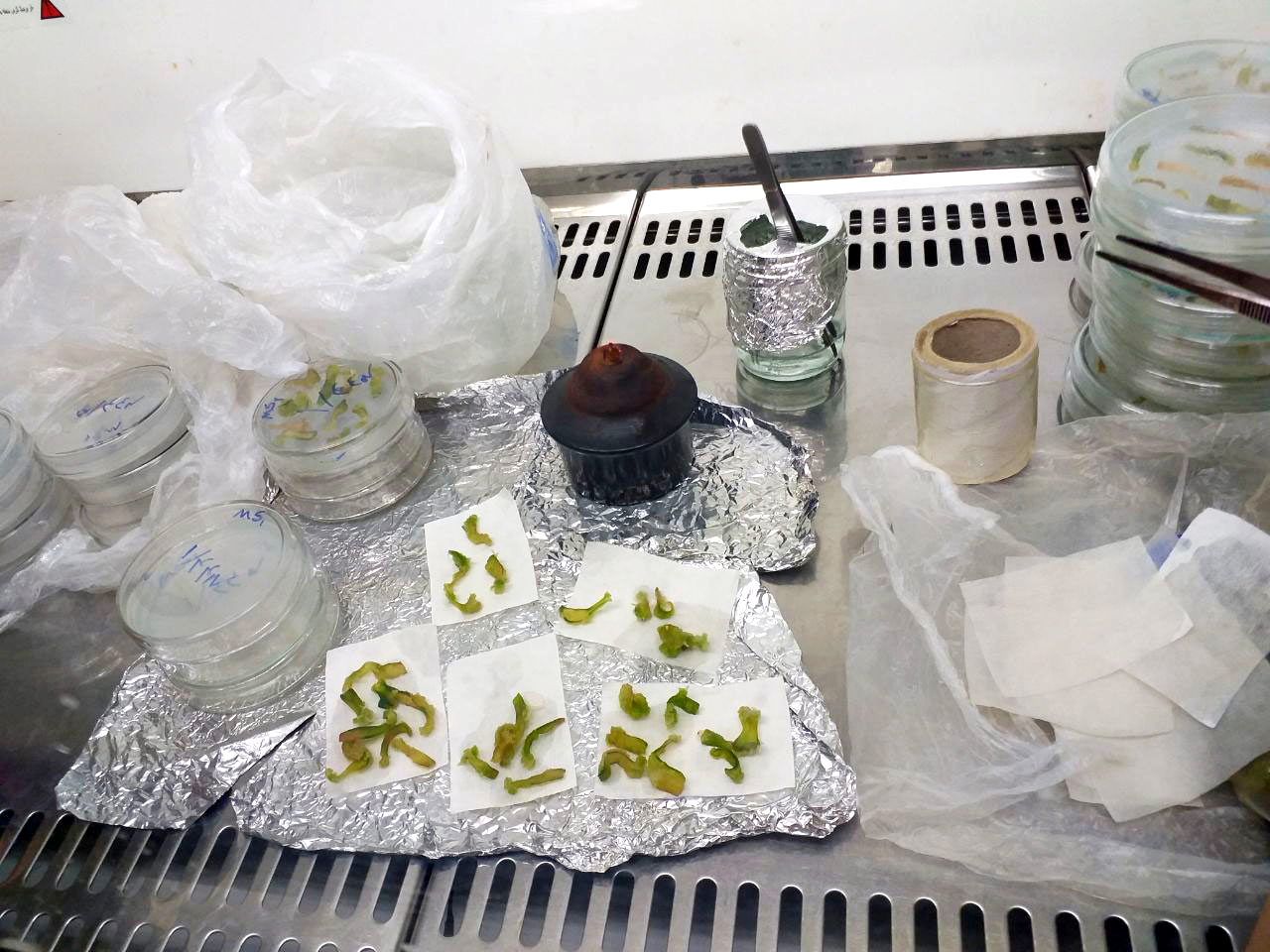 | 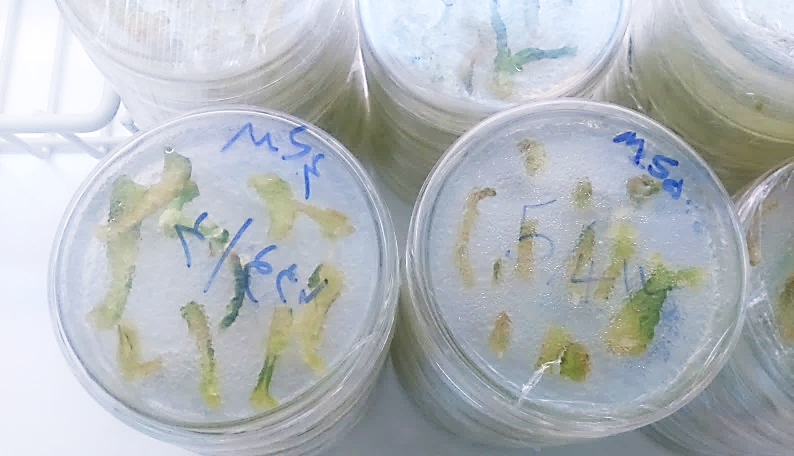 | 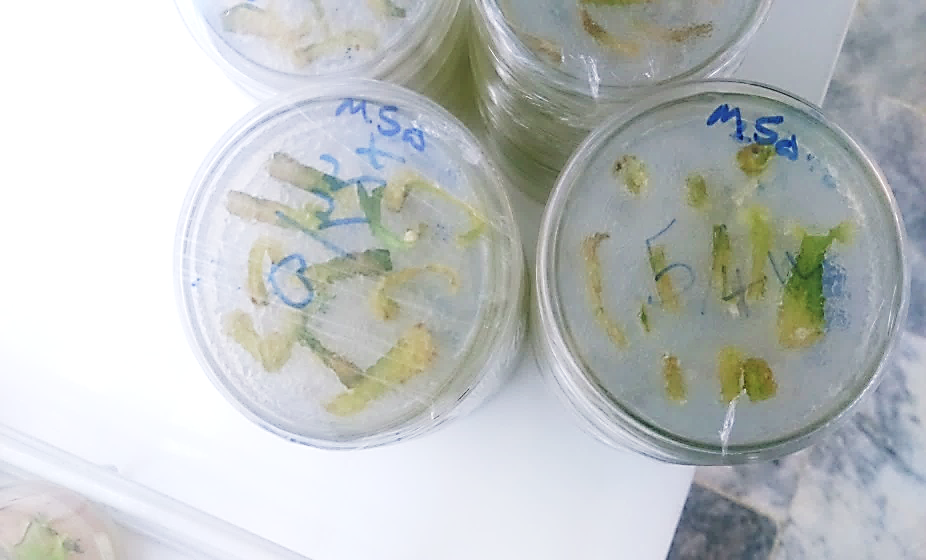 |
| --- | --- | --- |
| Inoculation of explants with *Agrobacterium* *tumefaciens* | Explants in co-cultivation media | |

| 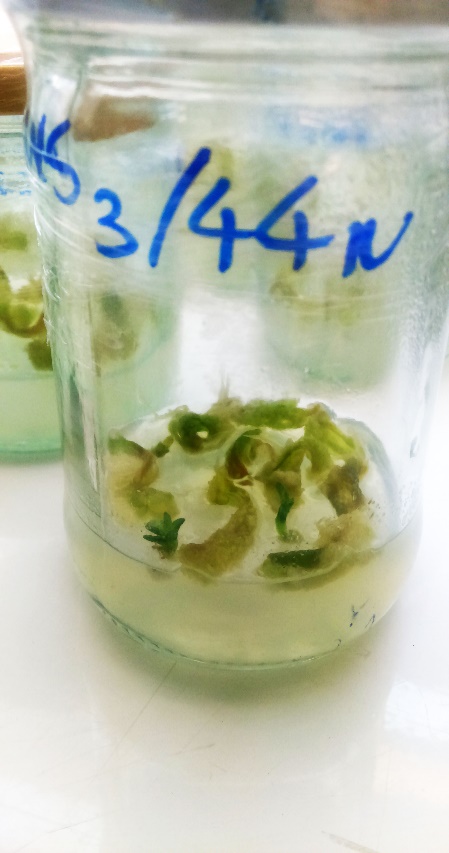 | 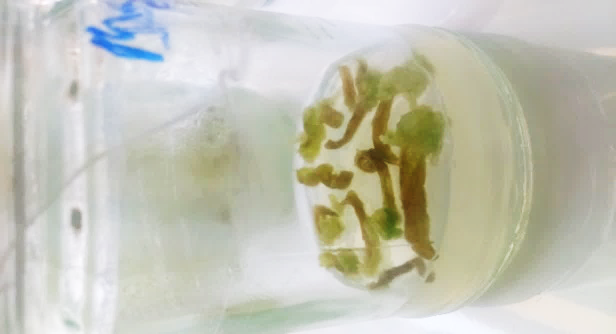 | 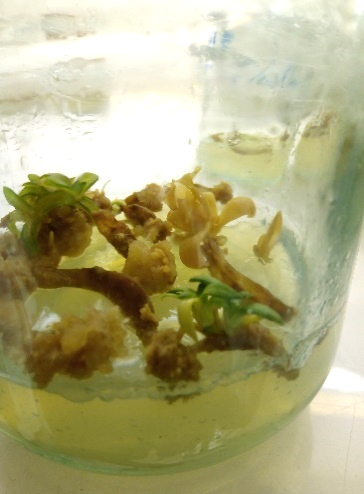 | 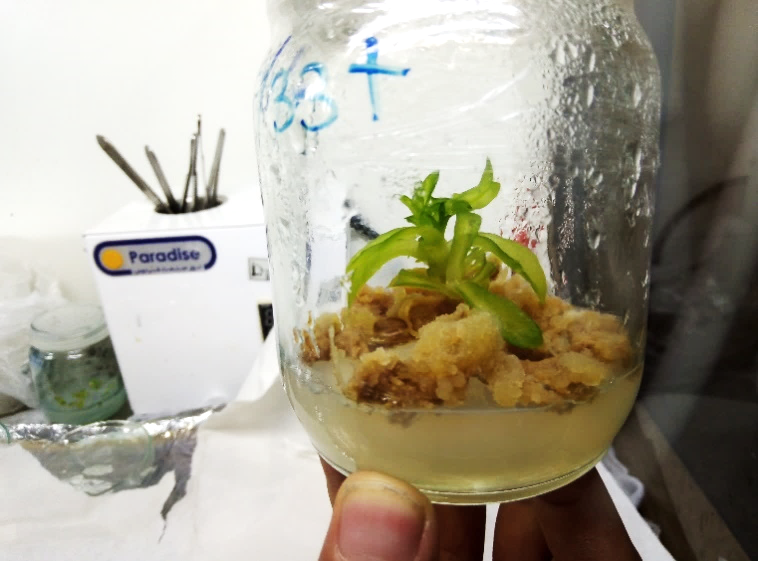 |
| --- | --- | --- | --- |
| 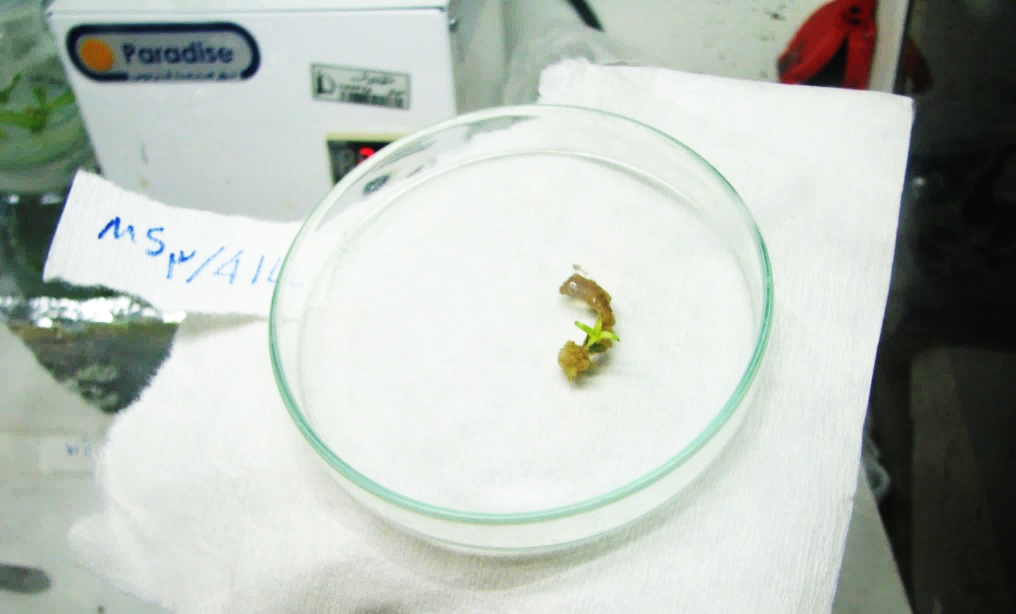 | 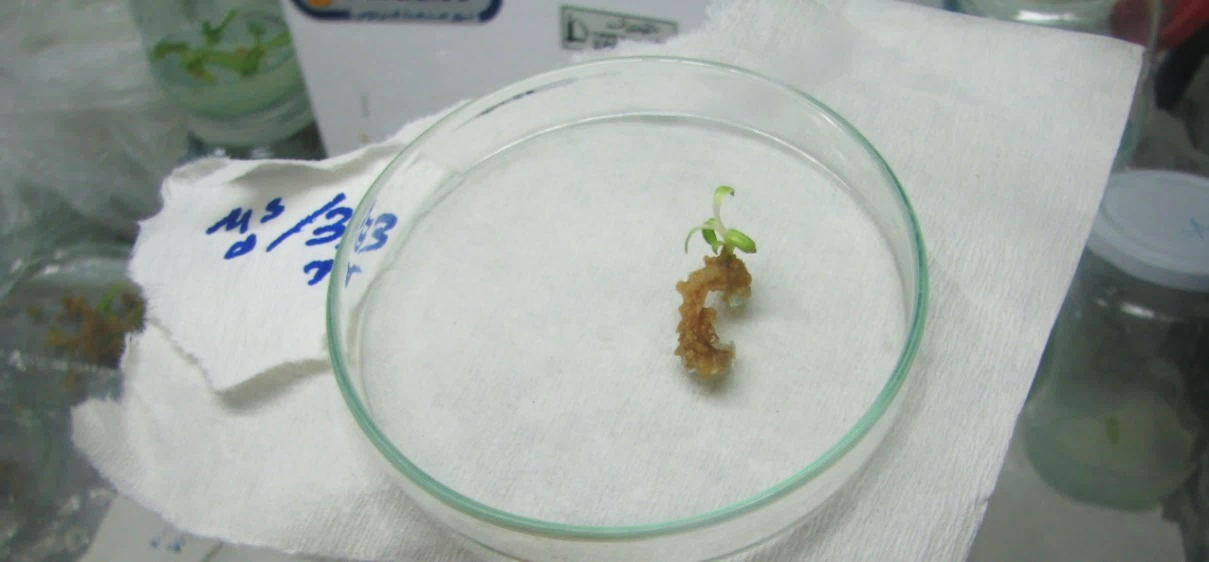 | 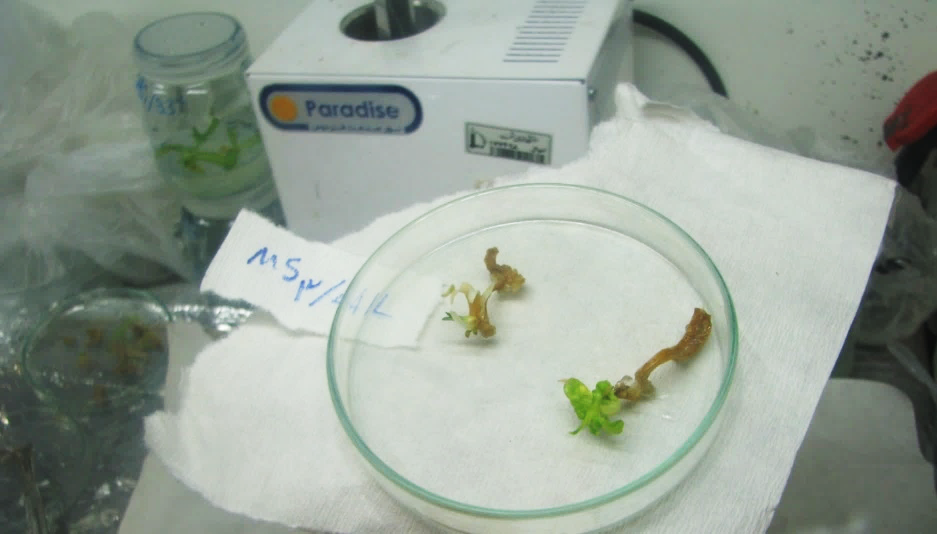 | 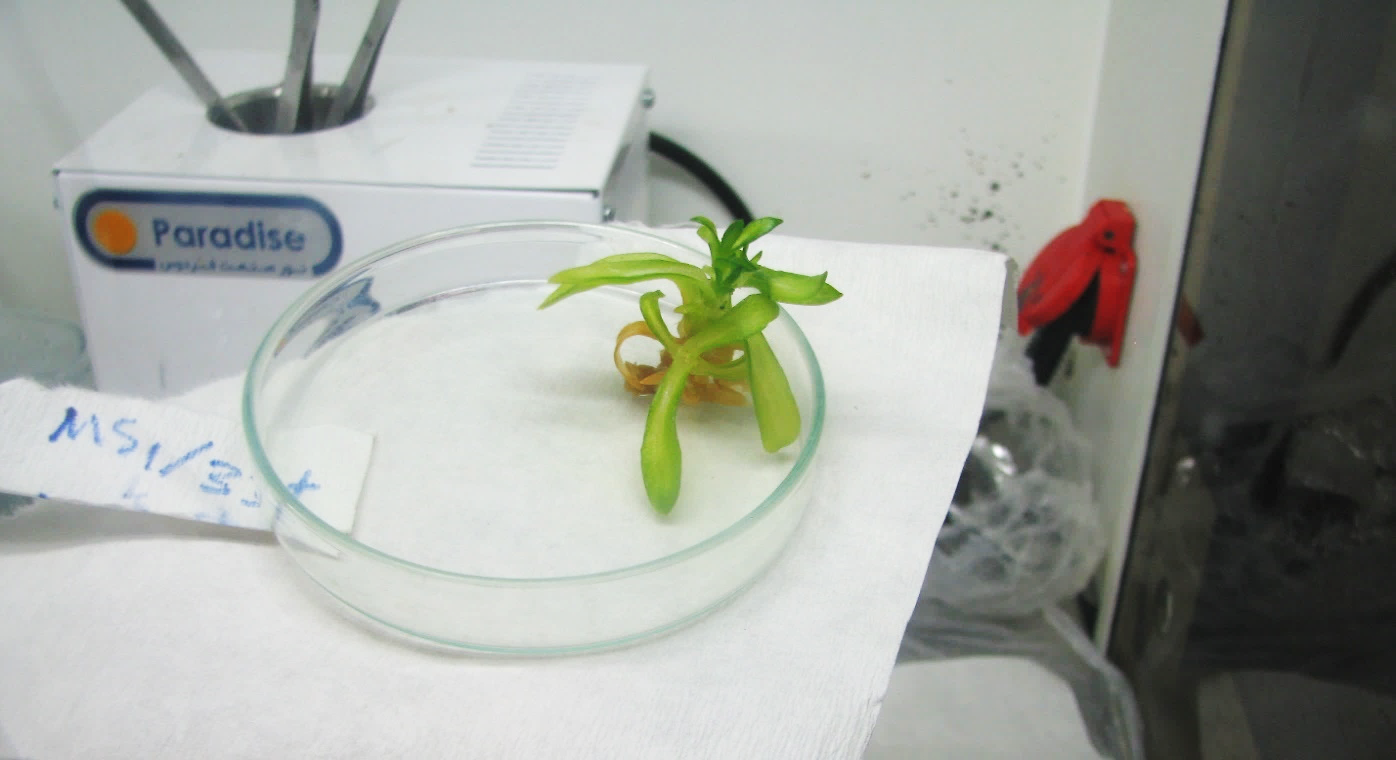 |
| Shoot regeneration from transformed calli in modified MS media | | | |

| 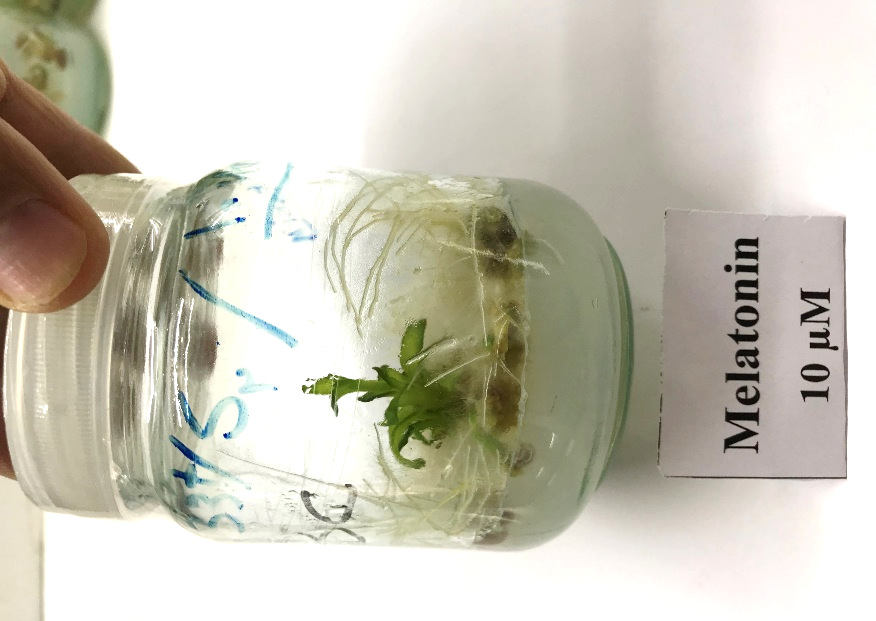 | 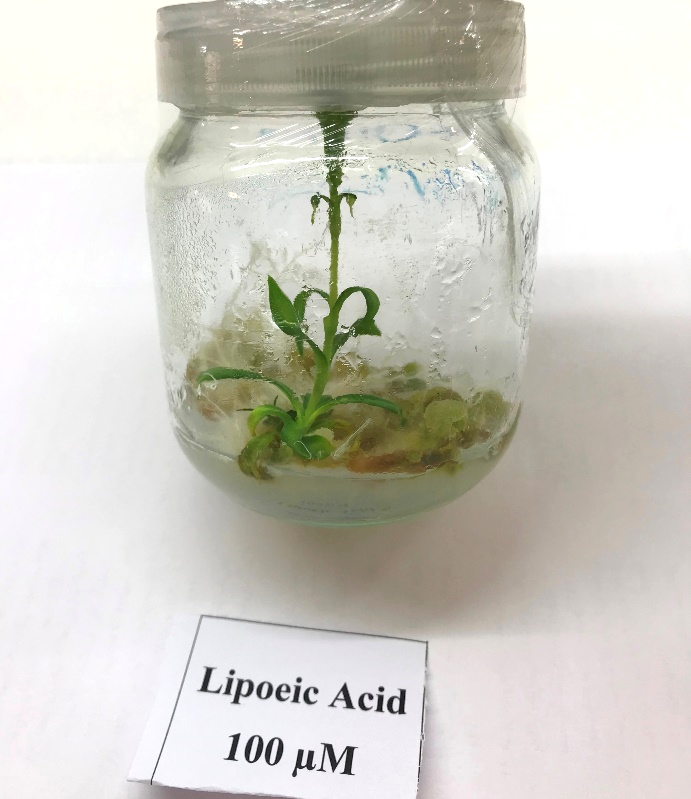 | 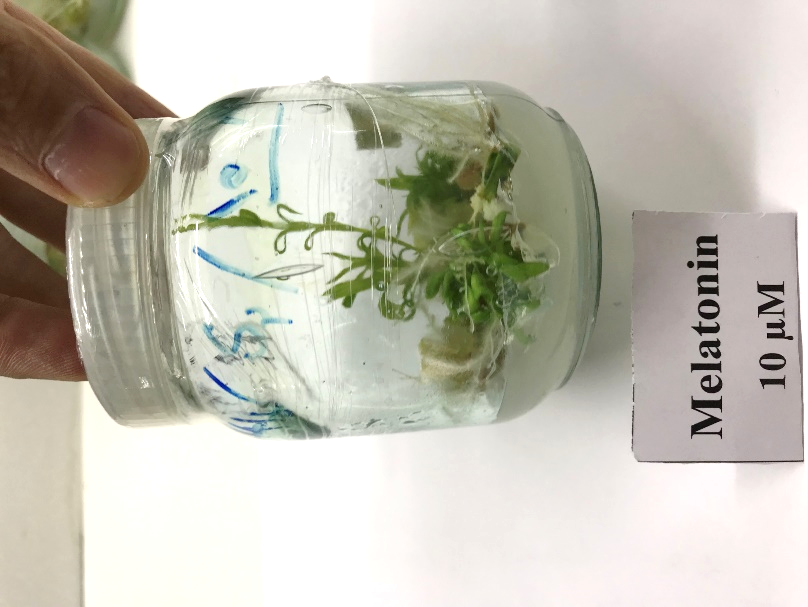 | 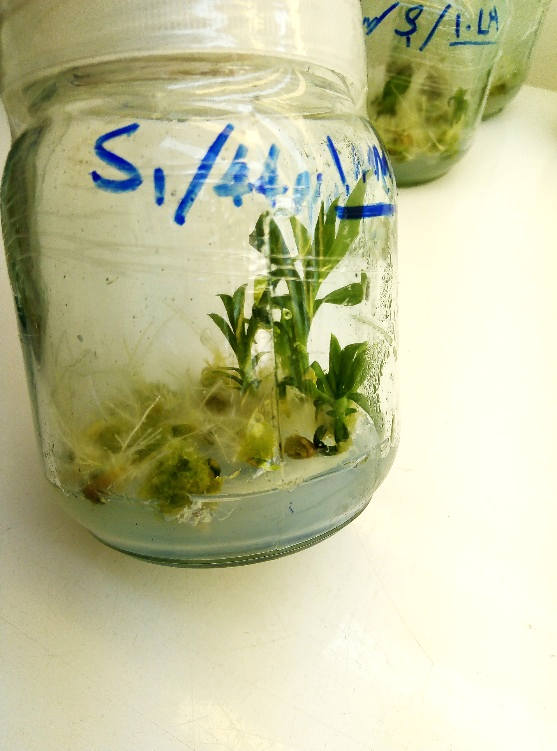 |
| --- | --- | --- | --- |
| 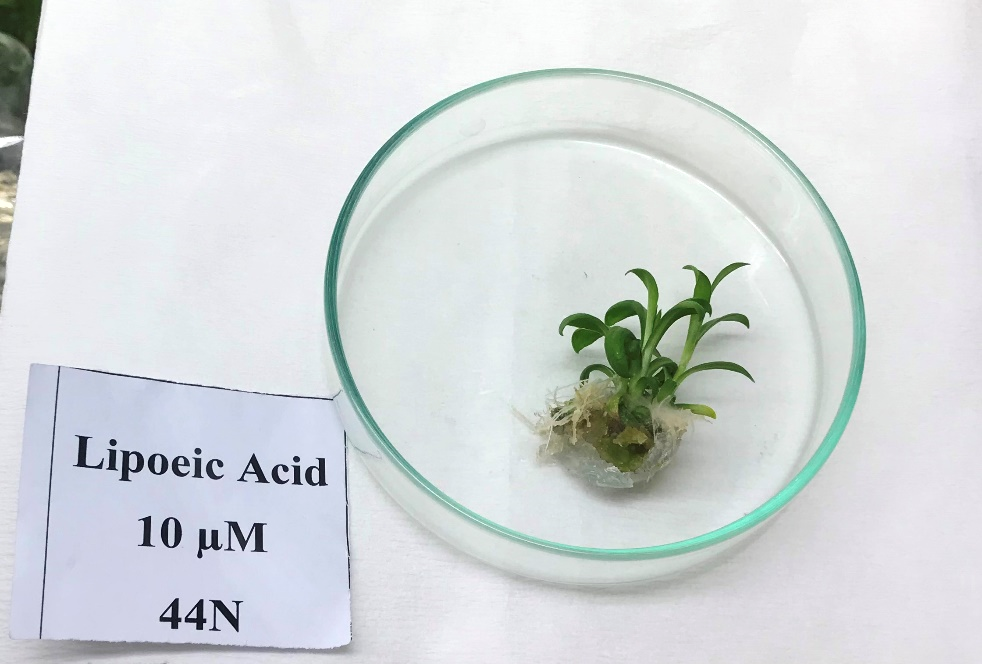 | 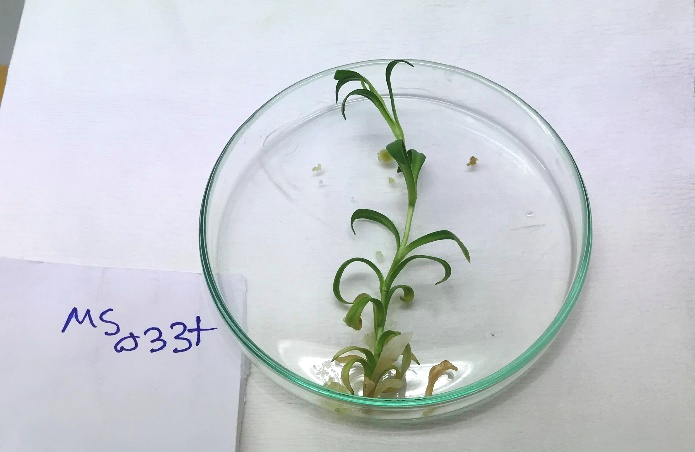 | 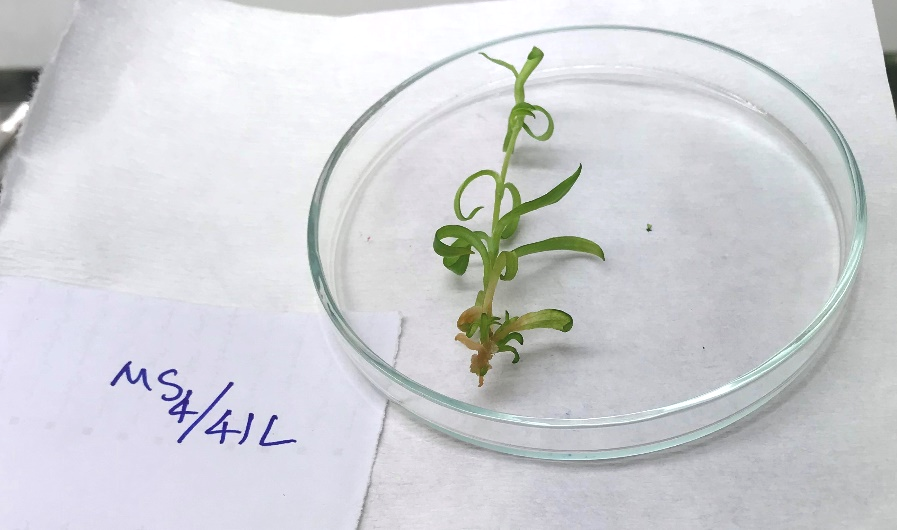 | 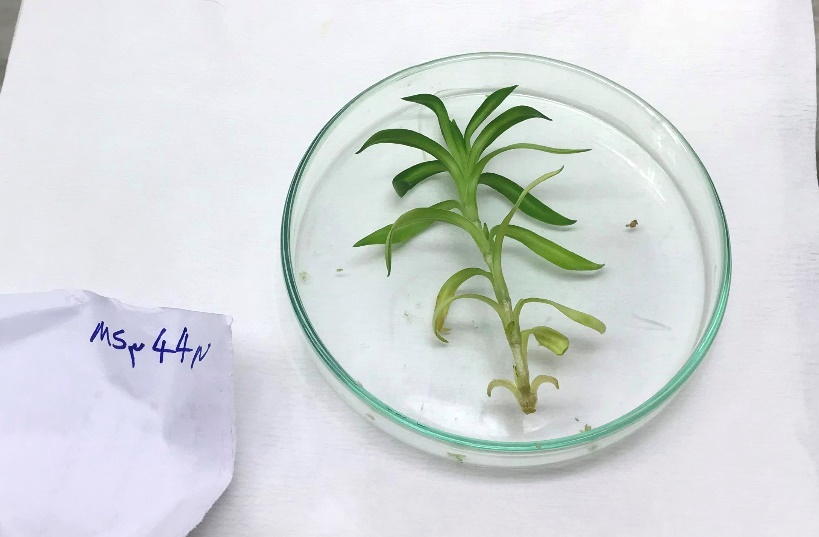 |
| Shoot regeneration from transformed calli in media containing α-lipoic acid or melatonin | | | |

| 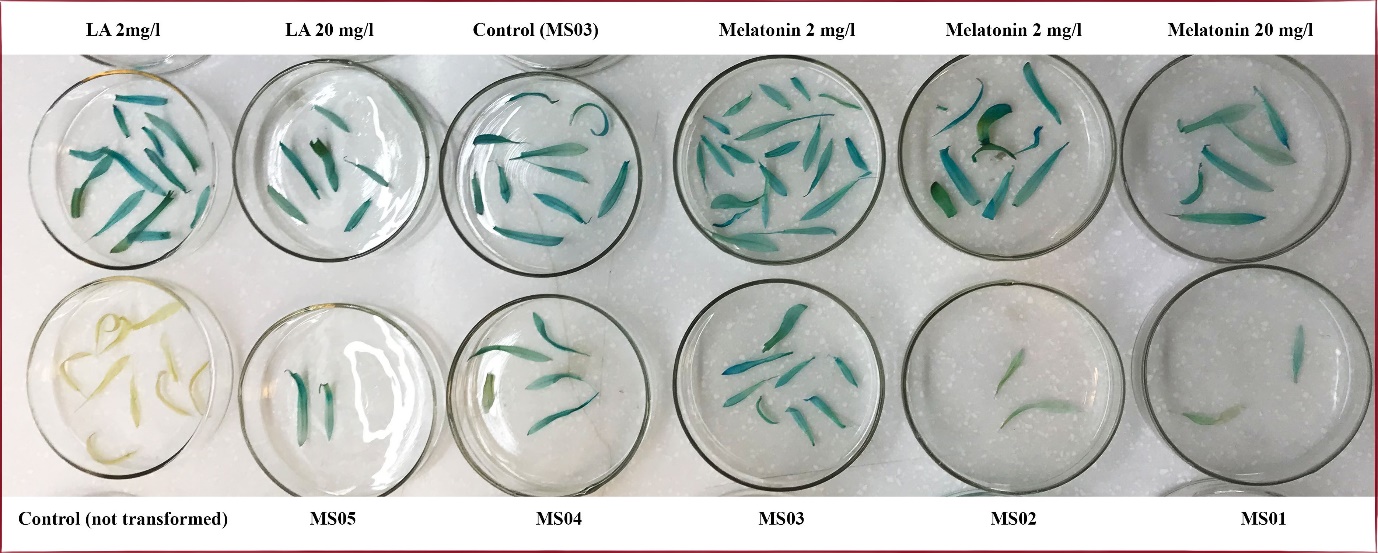 |
| --- |
| Histochemical GUS assay of the leaves obtained from transformed shoots, regenerated from callus |

| 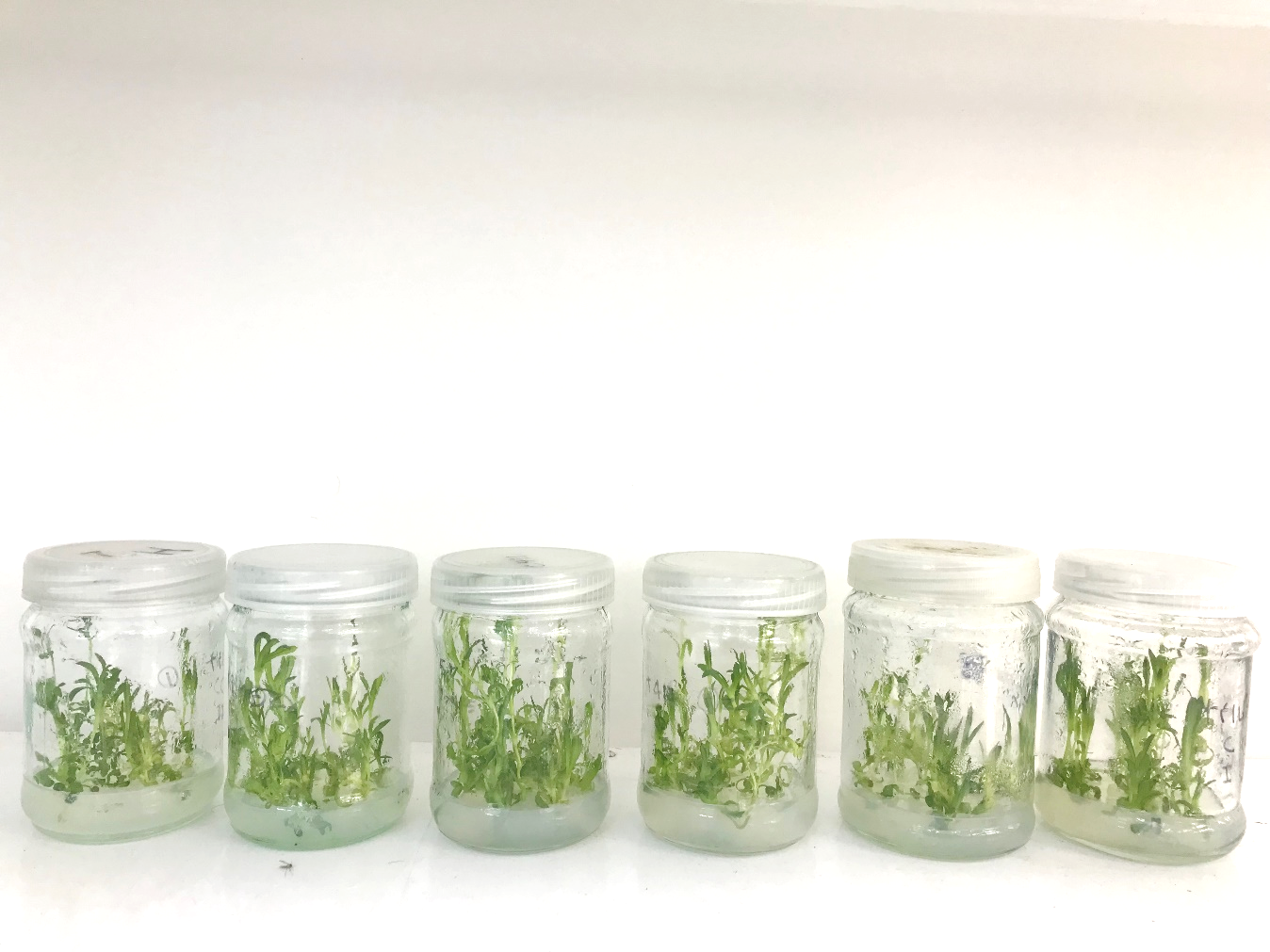 |
| --- |
| Shoot elongation of the transformed plants |

| 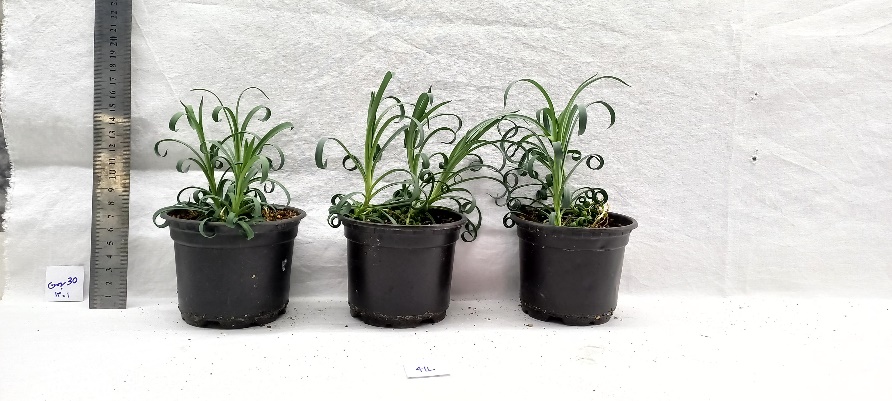 | 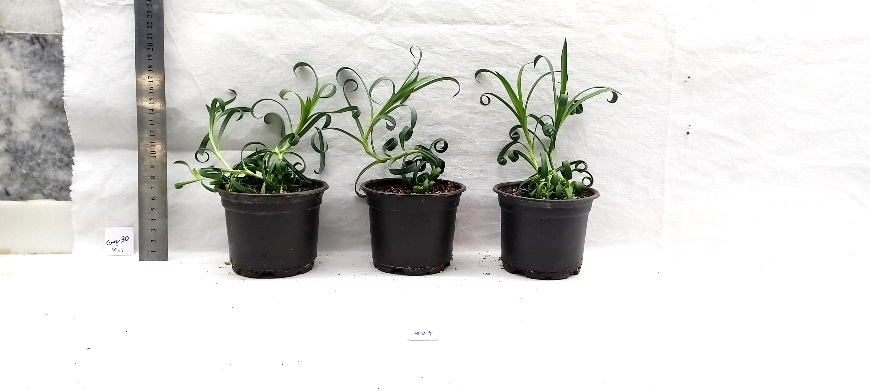 | 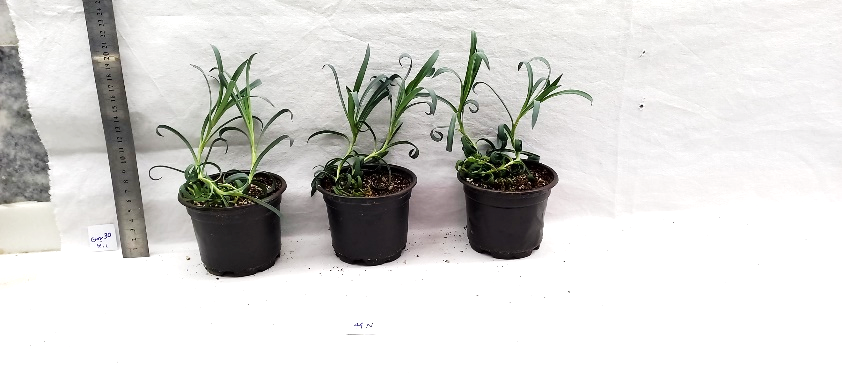 |
| --- | --- | --- |
| Acclimatized transformed plants after 3 months of transfer to ex vitro condition | | |
